# Focal osteoporosis defects play a key role in hip fracture

**DOI:** 10.1016/j.bone.2016.10.020

**Published:** 2017-01

**Authors:** Kenneth E.S. Poole, Linda Skingle, Andrew H. Gee, Thomas D. Turmezei, Fjola Johannesdottir, Karen Blesic, Collette Rose, Madhavi Vindlacheruvu, Simon Donell, Jan Vaculik, Pavel Dungl, Martin Horak, Jan J. Stepan, Jonathan Reeve, Graham M. Treece

**Affiliations:** a Department of Medicine, University of Cambridge and Addenbrooke's Hospital, Hills Road, Cambridge, UK; b Department of Engineering, University of Cambridge, Cambridge, UK; c Medicine for the Elderly, Addenbrooke's Hospital, Hills Road, Cambridge, UK; d Department of Orthopaedics, Norfolk & Norwich University Hospital, Norwich, UK; e Department of Orthopaedics, Faculty of Medicine, Charles University and Bulovka Hospital, Prague, Czech Republic; f Department of Radiology, Homolka Hospital, Prague, Czech Republic; g Faculty of Medicine 1, Charles University and Institute of Rheumatology, Prague, Czech Republic; h BOTNAR Research Institute, Nuffield Department of Orthopaedics, Rheumatology and Musculoskeletal Sciences University of Oxford, UK

**Keywords:** Osteoporosis, Pathogenesis, Hip fracture, Fracture prediction

## Abstract

**Background:**

Hip fractures are mainly caused by accidental falls and trips, which magnify forces in well-defined areas of the proximal femur. Unfortunately, the same areas are at risk of rapid bone loss with ageing, since they are relatively stress-shielded during walking and sitting. Focal osteoporosis in those areas may contribute to fracture, and targeted 3D measurements might enhance hip fracture prediction. In the FEMCO case-control clinical study, Cortical Bone Mapping (CBM) was applied to clinical computed tomography (CT) scans to define 3D cortical and trabecular bone defects in patients with acute hip fracture compared to controls. Direct measurements of trabecular bone volume were then made in biopsies of target regions removed at operation.

**Methods:**

The sample consisted of CT scans from 313 female and 40 male volunteers (158 with proximal femoral fracture, 145 age-matched controls and 50 fallers without hip fracture). Detailed Cortical Bone Maps (c.5580 measurement points on the unfractured hip) were created before registering each hip to an average femur shape to facilitate statistical parametric mapping (SPM). Areas where cortical and trabecular bone differed from controls were visualised in 3D for location, magnitude and statistical significance. Measures from the novel regions created by the SPM process were then tested for their ability to classify fracture versus control by comparison with traditional CT measures of areal Bone Mineral Density (aBMD). In women we used the surgical classification of fracture location (‘femoral neck’ or ‘trochanteric’) to discover whether focal osteoporosis was specific to fracture type. To explore whether the focal areas were osteoporotic by histological criteria, we used micro CT to measure trabecular bone parameters in targeted biopsies taken from the femoral heads of 14 cases.

**Results:**

Hip fracture patients had distinct patterns of focal osteoporosis that determined fracture type, and CBM measures classified fracture type better than aBMD parameters. CBM measures however improved only minimally on aBMD for predicting any hip fracture and depended on the inclusion of trabecular bone measures alongside cortical regions. Focal osteoporosis was confirmed on biopsy as reduced sub-cortical trabecular bone volume.

**Conclusion:**

Using 3D imaging methods and targeted bone biopsy, we discovered focal osteoporosis affecting trabecular and cortical bone of the proximal femur, among men and women with hip fracture.

## Highlights

• Hip fractures involve distinct patterns of focal osteoporosis in the proximal femur.
• We found less cortical and trabecular bone in key areas in those with acute hip fracture.
• Focal osteoporosis determines fracture location (femoral neck or trochanteric).
• Defects were detected using clinical CT scans of patients.

## Graphical abstract

**Figure:**
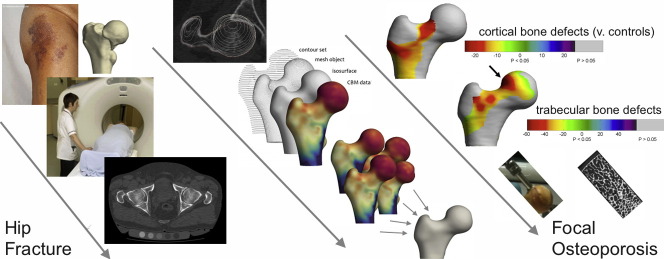

## 1 Introduction

Hip fractures are the most common reason for orthopaedic hospital admission in older people [1], [2], accounting for > 85,000 admissions annually in the UK [3] and causing considerable mortality [4]. > 90% of all hip fractures in the elderly are sustained through a fall, although some involve less trauma, happening spontaneously or during trips and stumbles [1]. Fracture occurs when mechanical strain is disproportionately concentrated on one part of the bone compared to surrounding regions [5]. The forces that act on a femur during floor-impact or during a stumble can be replicated in the laboratory. These simulations indicate that hip fractures initiate in small highly consistent focal regions of the proximal femur [6], [7]. In older adults, those regions have often become thin and porous through decades of age-associated bone loss, since they are stress-shielded during activities such as walking [8]. Micro CT measurements of trabecular bone from femoral head and neck biopsies support this notion, with substantially more bone present in areas directly loaded by walking than in fracture-prone zones [9], [10].

**Fig. 1:**
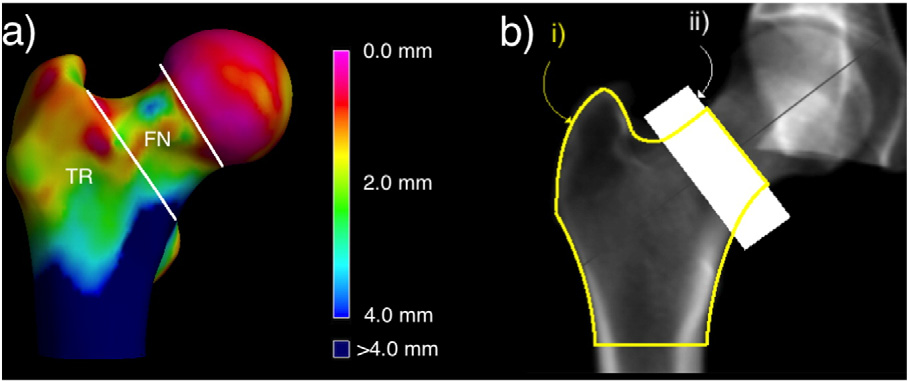
a) Boundaries for classifying the two main types of hip fracture; femoral neck (FN) and trochanteric (TR), shown on a 3D Bone Map of cortical thickness from a clinical CT scan b) Standard clinical two-dimensional areal bone mineral density (aBMD) measurements of i) the total hip region and ii) the femoral neck region. Approximate boundaries for aBMD measurement used in this study (QCTpro CTXA version 5.1.3, Mindways Software Inc., Austin, Texas).

Ordinary clinical computed tomography (CT) scans have been be used to identify focal 3D bone loss in ageing and as a predictor of fracture [11], [12], [13], [14], [15], [16]. Cortical Bone Mapping (CBM) couples CT imaging capability with an evaluation method to find average differences between groups known as statistical parametric mapping (SPM) [11], [12], [13], [14], [15], [16]. CBM can also be used to visualise femoral cortical thickness in CT scans from individual patients (Fig. 1).

By studying the intact contralateral femur of women with hip fracture from the Czech Republic (taking advantage of the anatomical similarity with the fractured side) we previously used CBM to discover thumbnail-sized patches of cortical thinning in fracture-prone focal regions [17]. Next we applied CBM to baseline scans from a case-cohort study of 288 healthy older male volunteers from the prospective MrOS study. Comparing the 99 men from the cohort who went on to sustain hip fractures with their fracture-free counterparts, we identified focal osteoporosis (focal regions of thin cortical bone [17] along with larger trabecular defects [18]) that predicted fracture (femoral neck, FN or trochanteric, TR Fig. 1a) slightly better than did two-dimensional areal bone mineral density (aBMD) measurements from Dual Energy X-ray Absorptiometry (DXA). Not only are the two hip fracture types known to originate at anatomically distinct foci (Fig. 1) [19] but from a surgical perspective are managed differently. Even though the overall prediction of hip fractures using CBM and DXA was similar [20], identifying focal osteoporosis through imaging techniques such as CBM might help in the proactive prevention of hip fracture given that targeted implantation of locally acting pharmaceuticals via the greater trochanter is technically feasible [21]. Traditional QCT measures of femoral neck and ‘total hip’ areal Bone Mineral Density (for example CTXA™, Mindways Austin, Texas, USA [22]) are now in routine clinical usage for hip fracture prediction by incorporation in the WHO FRAX™ tool. Several important questions remain; i) is focal osteoporosis present in the cortical and trabecular bone of women with hip fracture, and ii) do the CBM measures discriminate hip fractures better than traditional QCT measures of femoral neck and ‘total hip’ density (Fig. 1b)? To answer these questions we analysed data from several case-control studies, involving participants from both sexes who had sustained the two commonest types of hip fracture. We applied CBM [13], [14], [23] to clinical CT scans from the intact hip of cases and controls to define the 3D regions of interest (ROIs) where cortical and trabecular bone defects are associated with hip fracture, and then compared CBM with CTXA measures using Receiver Operator Characteristic analysis (ROC). Having identified defects, we then took targeted core biopsies from those regions at hemi-arthroplasty for accurate assessment of trabecular bone volume.

## 2 Materials and methods

### 2.1 FEMCO study; ethics and overview

The FEMCO study (Table 1) commenced in 2007, recruiting participants from Cambridge (LREC 07/H0305/61). Clinical CBM and CTXA measurements were made on the intact hip of fracture-patients scanned in an acute setting before surgical repair. After formal protocol amendments, the FEMCO study was adopted onto the UK National Institute for Health Research (NIHR) portfolio and several other centres contributed participants after informed consent (http://public.ukcrn.org.uk/search/StudyDetail.aspx?StudyID=5290). The study protocol, CT imaging parameters and recruitment were closely aligned with previous Cambridge studies (MRC-Hip Fx, MRC-Ageing LREC 06/Q0108/180, LREC99/076 and Anglo-Cardiff Collaborative Trial, ACC LREC 04/Q0108/257) as well as Prague studies (Study of Hip Joint in Trauma, IRB0002384101) [24]. All hip fractures were classified by the Muller AO criteria by a consultant radiologist (TDT) into femoral neck, FN (trans-cervical AO31-B1, B3 and sub-capital AO31-B2), or trochanteric, TR, types from multi-planar reformatted CT images. We combined fracture and control participants from all our studies in the present analysis. The key protocol details, including selection criteria for the FEMCO, MRC, ACC and Prague studies, are listed in Table 1, Table 2, Table 3, Table 4, Table 5.

### 2.2 Subjects and specimens

1 Femoral clinical CT scans from cohorts of patients; combined case-control studies
  1.1 Hip fracture patients (*n* = 70, 50 Female, 20 Male) versus 70 healthy gender matched controls
  1.2 Hip fracture patients (*n* = 138, Female) grouped by hip fracture type (52 Trochanteric, 86 Femoral Neck) versus healthy age and gender matched controls (*n* = 125)
  1.3 Frail patients with at least one injurious fall (*n* = 50) versus 50 healthy age and gender matched controls
2 Micro CT in hip fracture specimens
  2.1 FN hip fracture operative specimens (*n* = 14)

### 2.3 Description of studies

#### 2.3.1 Study 1.1

This case-control study used CBM and SPM to identify differences in bone parameters (cortical mass surface density CMSD and endocortical trabecular density ECTD) between 70 FEMCO patients with hip fracture (50F, 20M) and 70 gender-matched healthy controls from previous Cambridge studies [24], [25]. Our aim was to determine if the focal osteoporosis that we had identified in hip fracture patients from the Czech Republic [17] was present in an independent population.

#### 2.3.2 Study 1.2

This case-control study used CBM and SPM after combining women with hip fracture from FEMCO and Prague studies (*n* = 138, 52 Trochanteric and 86 Femoral Neck by Muller AO classification [26]) and comparing their bone parameters with those from 125 healthy age-matched female controls from previous Cambridge and Prague studies. Our aim was to determine if patches of focal osteoporosis differed between fracture types (TR or FN). A further aim was to determine how well CBM measures (CMSD and ECTD) with or without areal bone density measurements (CTXA) discriminated hip fractures from controls with Receiver Operating Characteristics (ROC) analysis. Protocol amendments were made to both FEMCO and the associated Prague Hip Joint in Trauma studies [17] to align selection and CT scanning criteria aiming to combine patients from both centres. This gave sufficient numbers of hip fracture cases and controls from each country to allow us to study differences between FN and TR fracture in women.

#### 2.3.3 Study 1.3

This case-control study used CBM and SPM to identify differences in bone parameters between 50 FEMCO female hospitalised fallers and 50 age-matched healthy female controls from previous Cambridge studies. Our aim was to determine if focal osteoporosis was present in fall-prone elderly women. Fallers were defined as having had a recent admission to an ortho/geriatric unit following a fall, with or without any fracture (except hip fracture), from a standing height or less.

#### 2.3.4 Study 2.1

This observational study involved targeted evaluation of the trabecular bone microstructure in the femoral head-neck junction (informed by the above studies) and another unloaded part of the femoral head, conducted at micro-CT resolution in 14 surgical specimens from FEMCO FN hip fracture patients. Our aim was to determine if focal osteoporosis could be identified by Bone Volume/Tissue Volume (BV/TV) measurement in affected areas.

### 2.4 Bone measurements; estimating cortical mass surface density and trabecular density from CT data

The analysis of local cortical parameters, such as thickness or cortical mass surface density, has historically been limited since thin structures such as the femoral cortex are not accurately depicted in clinical CT due to the limited spatial resolution of the images. Recent developments allow the estimation of local cortical and trabecular parameters from CT images. In this, as in previous studies, estimations were made at thousands of locations (‘vertices’) on a surface mesh of the unfractured proximal femur [13], [14]. The mesh was created by segmentation of femoral bone from surrounding tissues using Stradwin (v5.1 available free to download at http://mi.eng.cam.ac.uk/~rwp/stradwin). Also using Stradwin, the CT data was sampled at each vertex of the mesh using 18 mm lines perpendicular to and passing through the femoral cortex and trabeculae as described in detail recently [27]. Previously, we have used restrictive models of both the femur being scanned and the imaging system and then fitted these models to the observed data. Since a dense, thin, blurred cortex might appear identical to a less dense, thicker, blurred cortex with such an approach, we therefore needed to incorporate some prior knowledge about either the density or the blur; finding that assuming a specific, fixed value for the density was more successful than assuming a constant blur [14]. Later, we described a robust method for estimating this density, and extended the analysis to include cortical mass surface density which is the product of cortical thickness and cortical density (CMSD mg/cm^2^) as well as cortical thickness (CTh) [13]. We chose CMSD for the current analysis since it was recently found to be the most accurate and precise of the measures from cortical bone mapping [23]. Recognising the importance of trabecular bone measures in fracture discrimination, we also estimated endocortical trabecular density (ECTD, mg/cm^3^) by taking the calibrated height of that part of the 18 mm line passing through the trabecular bone. The process of registering femurs to a canonical (‘average’) surface yielded data on shape and bone size. Most (81%) of the variation in shape between femurs can be explained by a small number of deformation modes (as Whitmarsh et al. found [28], [29]). We therefore summarised each individual's shape as a 5-element vector representing the deformation of the femur along each of the 5 principal modes. The first mode could be described as overall femur size and the second approximates to femoral neck axis length. This 5-element shape vector was used in subsequent SPM. To measure aBMD, Mindways QCTpro CTXA (version 5.1.3, Mindways Software Inc., Austin, Texas) was used to give measures of femoral neck (Fn) or total hip (Th) aBMD in g/cm^2^ (as utilised in the current version of the WHO FRAX tool).

### 2.5 Statistical analysis models for SPM

Demographic variables were compared and expressed as mean and standard deviation with Student's *t*-test to determine the significance of any differences between cases and control (JMP v11, SAS institute). Statistical analysis of the registered femurs was performed using SPM [30] as implemented in the SurfStat package [31]. SPM involved fitting a generalised linear model to determine CMSD (or ECTD) in terms of explanatory and confounding variables. The model coefficients were then examined to reveal how the data depended on each covariate to identify where on the femur, if anywhere, each covariate was predictive of thickness or mass (with an associated *p*-value). Central to successful inference using SPM was the question of which covariates to include in the model. The null hypothesis was that there would be no focal differences in CTh, CMSD or ECTD between fracture and controls in 3D femoral structure. Therefore the fixed effects generalised linear SPM model (GLM) used for Study 1.1 was CMSD (or ECTD) = 1 + group + age + weight + sex + shape 2 + shape 3 + shape 4 + shape 5. The same GLM, but substituting site (Czech Republic or UK) for sex was used for Study 1.2. Group was defined as ‘fracture or control’ in Study 1.1, ‘FN fracture, TR fracture or control’ in Study 1.2 and ‘faller or control’ in 1.3. In these models, age and weight, site and shape modes 2–5 were modelled as confounding variables. Allowing for shape (parameterized using the discrete shape modes derived from the registration process) guarded against false inference caused by systematic misregistration [32]. We did not model shape mode 1 (femur size) or patient height, since these covariates were correlated with group and we would have encountered the well-known problem of correlated regressors. Consequentially, we would have been in danger of missing significant effects [31] because the statistical tests in SPM reveal only the variance that can be ascribed uniquely to the covariate(s) of interest [33], [34]. The only imaging-derived explanatory variables were the shape modes, and there was no justifiable reason to expect these to differ with study (and certainly not with scanner/phantom type), so group was not nested within ‘study’ in 1.2.

### 2.6 Creation of a region of interest (ROI) for discriminatory analysis (Receiver Operating Characteristics)

Single average measurements across the entire ROI (generated from the completed process of SPM for each patient in Study 1.2) were then derived. In the present study, there were four ROIs: a ‘femoral neck fracture cortical’ and a ‘trochanteric fracture cortical’ ROI as well as a ‘femoral neck fracture trabecular’ and ‘trochanteric fracture trabecular’ ROI (for ROI boundaries, see Fig. 3c–f). These single ‘patch average’ values were then taken forward (along with demographics) to test the discriminatory ability of CT bone mapping ROIs (CMSD or ECTD) to correctly classify fracture versus control and also TR and FN types. CBM was compared with CTXA. Receiver Operating Characteristics (ROCs) were created using leave-one-out cross-validation, such that predictions for each subject were only ever made on ROIs and models not containing that subject. Individual odds ratios for each ROI, as well as for CTXA values, were calculated separately from multinomial regression.

### 2.7 Precision measurements

Precision was previously assessed in repeat analysis of 19 individuals re-imaged with CT and re-analysed after an average of 61 days (standard deviation 22 days, range 34 to 107 days) giving a measurement precision of 5% of the mean at individual measurement points, dropping to 1% of the mean when measurements were averaged over a defined region of interest.

### 2.8 Bone measurements: femoral head surgical specimen selection and preparation

**Fig. 2:**
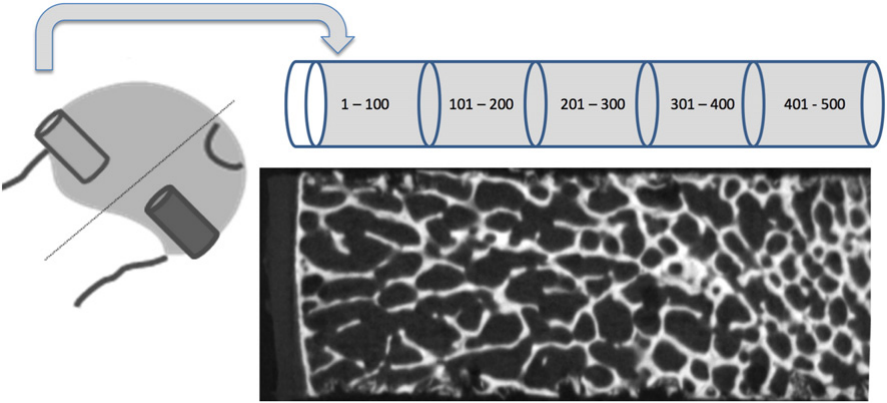
Biopsy regions. The cartoon shows the locations for the biopsies, and a resulting XTEK high resolution scan image through the femoral head of a FEMCO study participant who had donated their femoral head at operation. Also shown are the five 100-slice segments.

Femoral heads were obtained from 14 FEMCO participants who had undergone CT scanning prior to surgery. Once each femoral head had been removed at arthroplasty it was placed in 70% ethanol for at least 7 days for fixation. Femoral heads were sawn in half to make taking the cores easier. A modified Bordier trephine (diameter 7 mm) was used to take core biopsies from each half, one from the ROI in the anterolateral femoral head known to be associated with FN fracture [17] and the other from the subfoveal region on the opposite side of the head for comparison (Fig. 2).

Each biopsy was placed cortex down in a Perspex mould and scanned in air in a Metris X-TEK HMX CT 160 microCT scanner (Nikon Metrology, Derby, UK) at 50 kV and 60 μA, FOV 1024 × 1024 pixels, acquisition time c. 1 h. Images were centred and squared in XTEK – NGI control software then images were further straightened in CT PRO software (supplied with the scanner). VGStudio max software (Volume Graphics GmbH, Heidelberg, Germany) was used to create DICOM images (voxel size 0.02 × 0.02 × 0.02 mm). ImageJ > Bone J (http://imagej.nih.gov/ij/) was used to open the DICOM data (orientated perpendicularly to the long axis of the cylindrical biopsy, Fig. 2, lower pane) [35]. Images containing just the trabecular portion of the biopsy were then selected. The middle image of this stack was chosen as a reference point for each core for automatic brightness and contrast adjustment. On that same image a circular ROI was created (6.26 mm) leaving a small margin at the periphery of the core. The images were segmented into bone and background and an automatic measurement of bone volume/tissue volume (BV/TV) was taken using the method described by Doube [35] to give an overall BV/TV for the core. Using the ‘delete slice range’ option from the ‘stacks’ sub option of the ‘plugins’ menu BV/TV measurements were then averaged throughout the core in 100 slice stacks (2.3 mm) to determine BV/TV through the length of the biopsy from the subcortical part to the trabecular end of the biopsy (Fig. 2). This method involved reloading the stack of images before cutting it down to measure each 100-slice stack. Thus, internal quality control was provided as the whole biopsy BV/TV was measured each time and the coefficient of variation of the overall biopsy BV/TV ranged in individual cases from 0 to 0.76%. BV/TV data was complete for all cores for the first three hundred slices; after that point due to damage to the cores (caused by either the femoral head extraction process or crushing during the biopsy extraction process) the data were less complete so that only the first 500 slices (11.5 mm) could reasonably be compared. Paired t-tests were performed comparing each stack of 100 images between the two biopsy sites.

## 3 Results

### 3.1 Study 1.1

**Fig. 3:**
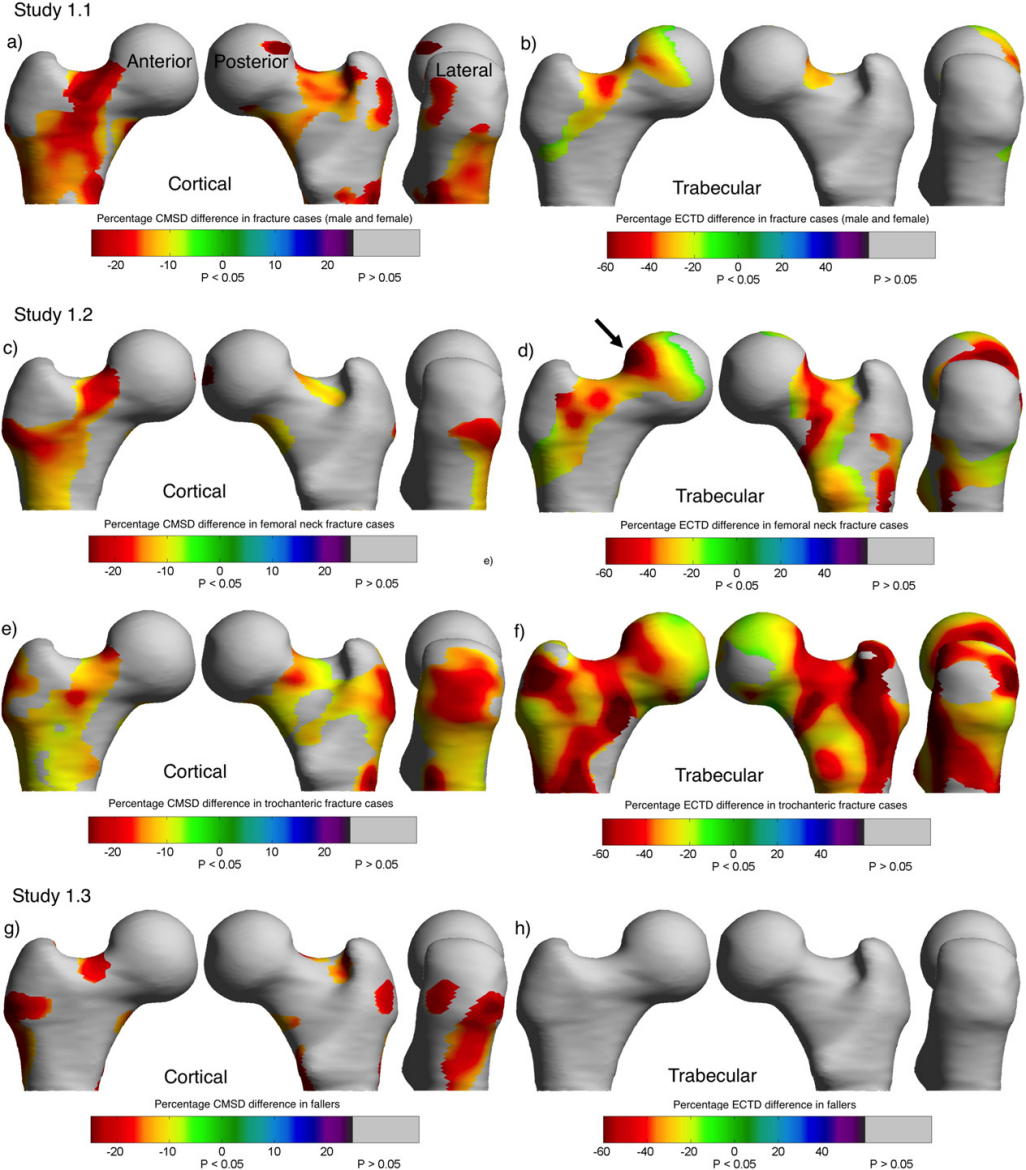
Study 1.1. Bone Mapping (CBM) ROIs. Statistically significant differences in cortical **(a)** and trabecular **(b)** bone between hip fracture (*n* = 70, 50 female, 20 male) and 70 healthy controls shown as a colour map on the canonical femur model. CMSD Cortical Mass Surface Density, ECTD Endocortical Trabecular Density Study 1.2. Cortical **(c)** and trabecular **(d)** differences between female femoral neck patients (*n* = 86) and controls (*n* = 125). Cortical **(e)** and trabecular **(f)** differences between female trochanteric fracture patients (*n* = 52) and controls (*n* = 125). The black arrow highlights the biopsy site for Study 2.1. Study 1.3. Cortical **(g)** differences between frail female patients with at least one injurious fall (*n* = 50) versus 50 healthy controls (no difference in trabecular bone **(h)**).

Comparison of 70 hip fracture patients (20 male and 50 female) with 70 healthy gender-matched controls: Fig. 3a and b show colour maps of the statistically significant differences between hip fracture patients and healthy controls in cortical mass surface density (CMSD, the primary outcome measure) and trabecular bone (ECTD). The focal differences involved several ROIs including the supero-anterior and inferior femoral neck. The hip fracture cases were statistically significantly older (cases mean age 78.3 yrs ± 9.9 standard deviation; healthy controls 74.0 ± 9.1, *p* = 0.0084), lighter (cases weight, 62.6 ± 12.5 kg; healthy control 70.9 ± 13.5, *p* = 0.0002) and had lower aBMD (cases femoral neck aBMD, 0.53 ± 0.11 mg/cm^2^, healthy control 0.70 ± 0.13, *p* < 0.0001). They were only well matched for height (cases height, 1.65 ± 0.09 m; healthy controls 1.64 ± 0.10, *p* = 0.74). There were 51 FN fractures, but only 19 TR fractures, hence the need to combine these subjects with the Prague Study of Hip Fracture participants (Study 1.2).

### 3.2 Study 1.2

Comparison of hip fracture type (FN, *n* = 86 or TR, *n* = 52) with healthy age and gender matched controls (all female, *n* = 125): The main finding was the strikingly focal, rather than generalised, difference in cortical and trabecular bone between controls and fracture cases (Fig. 3c–f). FN fracture types had a focal cortical bone defect that encompassed the superior femoral head neck junction and passed around the inferior neck and towards the anterior part of the trochanter. TR fractures also had a marked cortical bone defect in the trochanter itself. There was at least 20% less cortical bone in the ‘core’ of the ROI, compared with controls. Fig. 3d also shows a matching focal trabecular bone defect in FN fracture cases (similar to the cortical regions) and this was the site chosen for the surgical trephine biopsy taken for high-resolution microCT in Study 2.1 (shown by a black arrow). There was a more generalised trabecular bone deficit in TR fracture cases. Trabecular bone loss affected more of the femur than cortical bone loss in both fracture types. For femoral neck fractures, considering the total bone surface measured, 35.4% of trabecular and 18% of cortical measurements were statistically significantly lower than control. 22% of the areas with trabecular defects had corresponding cortical defects. For trochanteric fractures, on average 82.1% of trabecular and 48.9% of cortical measurements were lower than control. 36.9% of areas with trabecular defects had corresponding cortical defects. The combined UK and Czech female hip fracture cases were statistically significantly lighter (cases weight, 0.59 ± 0.13 kg; 63.2 ± 11.5 healthy control 66.4 ± 11.2, *p* = 0.024), taller (cases height, 1.62 ± 0.07 m; healthy controls 1.58 ± 0.07, *p* < 0.0001) and had lower femoral neck CTXA aBMD (cases aBMD, 0.59 ± 0.13 mg/cm^2^; healthy control 0.69 ± 0.12, *p* < 0.0001). They were well matched for age (cases age, 77.4 ± 8.7 yrs; healthy controls 76.8 ± 7.3, *p* = 0.56). FN cases and TR cases had similar femoral neck aBMD (FN fracture aBMD, 0.59 ± 0.13 mg/cm^2^; TR 0.60 ± 0.12, *p* = 0.53), age (FN age, 77.1 ± 8.8 yrs; TR 77.4 ± 8.9, *p* = 0.89) and weight (FN weight, 62.6 ± 10.2 kg; TR 63.6 ± 13.1, *p* = 0.61). Women with bigger femurs (larger shape mode 1) were more likely to have sustained hip fractures (FN difference in shape mode 1 v control + 0.77 ± 0.32, *p* < 0.0001, TR + 0.50 ± 0.31, *p* = 0.002). There were no differences between hip fracture and controls in any of the other shape parameters (*p* values ranged from 0.23 to 0.75).

### 3.3 Study 1.3

Comparison of 50 frail females who had fallen with 50 healthy age and gender-matched controls: Fig. 3g shows a statistically significant difference between UK FEMCO fallers and healthy controls in CMSD. The fallers had lower CMSD than controls in ROIs that were smaller, but in similar anatomical locations to those observed in the hip fracture cases (Study 1.1). Although this suggests that cortical bone defects are also present in frail fall-prone elderly women, there were no statistically significant differences in trabecular density (ECTD, Fig. 3h).

This result confirms that inpatient fallers are a potentially unsuitable control group for studies involving hip fracture patients, due to ascertainment (Berkson's) bias [36]. The female fallers were well matched to controls for age (fallers age, 75.5 ± 8.6 yrs.; healthy controls 77.3 ± 7.5, *p* = 0.28), weight (fallers weight, 69.1 ± 16.0 kg; healthy control 67.1 ± 12.1, *p* = 0.48) and height (fallers height, 1.61 ± 0.07 m; healthy controls 1.60 ± 0.07, *p* = 0.34). The fallers had statistically significantly lower femoral neck aBMD (fallers aBMD, 0.59 ± 0.13 mg/cm^2^; healthy control 0.65 ± 0.09, *p* = 0.0096).

### 3.4 Study 2.1

**Fig. 4:**
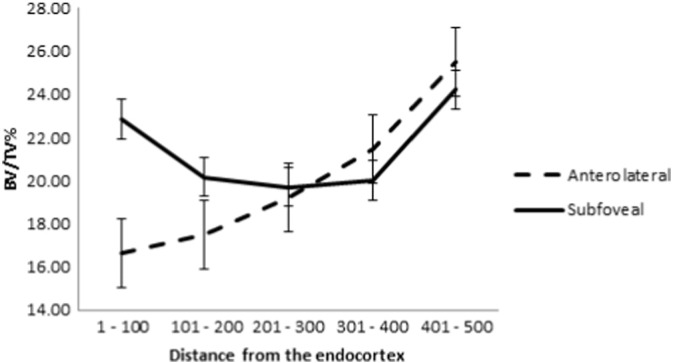
Results from biopsy study. The graph shows the statistically significantly lower BV/TV (with standard error of the mean) in the trabecular area highlighted by the Bone Mapping technique (black arrow in Fig. 3d). *p* = 0.046 for the paired difference between 1 and 100 slices.

Femoral neck hip fracture operative specimens: There was statistically significantly less sub-cortical trabecular bone volume (BV/TV) in the targeted biopsy taken from the ROI identified in Fig. 3d compared with the inferior subfoveal core (see Fig. 4). The surgical specimens came from individuals who represented the overall UK FEMCO sample well for age (biopsy patients age, 78.0 ± 8.5 yrs; all FEMCO hip fracture patients 78.6 ± 9.7, *p* = 0.57), height (biopsy patients height, 1.65 ± 0.10 m; all FEMCO 1.63 ± 0.09, *p* = 0.48) and weight (biopsy patients weight, 65.4 ± 17.7 kg; all FEMCO 61.3 ± 11.8, *p* = 0.47). The biopsy patients had similar femoral neck aBMD to the rest of the FEMCO cases (biopsy patients aBMD, 0.57 ± 0.12 mg/cm^2^; all FEMCO 0.52 ± 0.11, *p* < 0.07), and it is also noteworthy that their average T-score was not in the osteoporotic range –2.02 (± 1.04). Examining paired samples, the only significant difference was in the sub-cortical region of the cores (*p* = 0.046) beyond that point there were no significant differences.

### 3.5 Receiver operating characteristic (ROC) curves for fracture determination

**Fig. 5:**
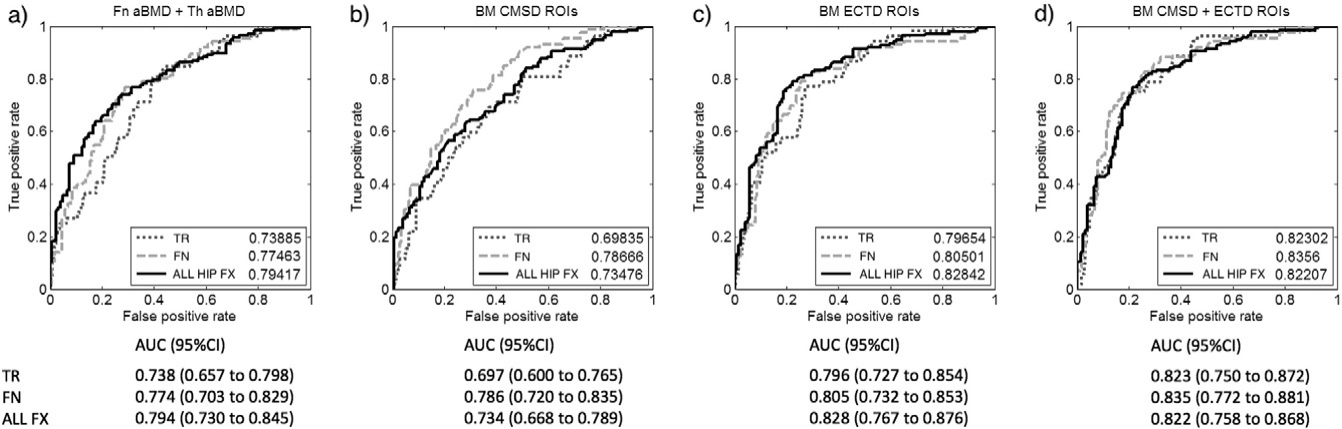
**a**) Area Under the Curve (AUC) from Receiver Operating Characteristic analysis for the ability of age, height, Femoral neck (Fn) and Total Hip (Th) DXA-like areal Bone Mineral Density (aBMD) from CT to correctly categorise hip fracture types (grey lines- dotted for TR or dashed for FN) as well as all hip fractures (black solid lines-ALL HIP FX). ROC analysis for different combinations of the novel 3D Cortical Bone Mapping (CBM) measures to correctly discriminate hip fractures (5b–d). An average single measure of 3D Cortical Mass Surface Density or Trabecular Density (ECTD) was taken for each patient from the bone mapping ROIs (shown as patches in Fig. 3c–f). The ability of age, height and an average 3D measure of either CMSD (**5b**), ECTD (**5c**) or both CMSD and ECTD (**5d**) to correctly discriminate fractures, as well as the corresponding AUC values and 95% confidence intervals for discriminating all fractures (ALL FX), Trochanteric fractures (TR) and Femoral neck (FN) are shown.

The results described in Study 1.2 led us to test individual parameters and combinations of parameters in sensitivity analyses. We calculated a single average cortical measurement from each patient incorporating values across all significant bone mapping ROIs shown in Fig. 3(c–f), repeating that process for trabecular bone ROIs. We calculated the Area Under the Curve (AUC) from Receiver Operating Characteristic (ROC) analysis to compare fracture discrimination using bone mapping (cortical, trabecular or both) values with aBMD of the femoral neck (Fn) and total hip (Th) regions. It can be seen from Fig. 5a, that Fn and Th aBMD together are good at discriminating all fractures from control subjects (AUC 0.79). aBMD is somewhat less effective at determining fracture type (dotted and dashed grey lines). Bone mapping measurements from cortical ROIs *alone* did not increase the AUC for discriminating fractures from controls (Fig. 5b). It was only after adding values from the trabecular ROIs that the AUC improved to be numerically greater than aBMD (AUC 0.82, Fig. 5c and d), with improved deviance statistics. While individual adjusted odds ratios do not quantify classification accuracy, we did find that odds ratios from multinomial regression were numerically greater for trabecular bone measures than they were for cortical bone measures (Table 6).

## 4 Discussion

Femoral neck and trochanteric fracture patients had different patterns of focal osteoporosis. Such focal osteoporosis is unfortunate, since it affects locations in the proximal femur where cracks are expected to initiate during falls (at least as simulated in the laboratory [6], [5]). Trabecular bone loss affected more of the femur than cortical bone loss in both fracture types. As reflected in ROC analysis and odds ratios the ability to effectively discriminate hip fractures from controls was better when including trabecular (as well as cortical) measurements. In our study, women with femoral neck fractures had large focal defects located within the superior neck, particularly at the head-neck junction (Fig. 3c–d), compared with controls. Women with trochanteric fractures lacked trabecular bone throughout the femur (except in the loaded inferior cortex), but also lacked cortical bone in a focal patch that incorporated both the lateral trochanter and superior femoral neck (Fig. 3e–f). In ROC analysis, CBM measures improved upon CTXA measures for discriminating fracture type from controls (from 0.79 with CTXA to 0.82 with CBM), but crucially, this was only when trabecular bone was included in the model. This adds considerable support to data indicating that trabecular bone is an important determinant of hip fracture [37], [38], [39], [40]. Deficient trabecular bone in the hip [41], [42], [43] has been implicated in increased fracture risk due to anisotropy leading to inability to cope with loading from an unexpected direction as in a sideways fall [44]. Since cortical thinning reduces the hip's capacity to absorb energy imparted by a fall [7], it follows that trabecular bone loss adds to this effect. By directly sampling the areas identified as most deficient by imaging (Fig. 3d, black arrow), our MicroCT findings confirm focal osteoporosis (“too little bone in the bone” [45]). The biopsied patients did not have generalised osteoporosis as evidenced by their aBMD T-scores, which underlines the focal nature of the bone volume defect found.

DXA scanning for fracture risk estimation is unlikely to be surpassed by higher radiation CT technology in routine clinical practice, but the present study is useful in confirming the clinical utility of both CTXA and CBM measurements. Our findings fit well with the work of Duboeuf and co-workers using the EPIDOS prospective cohort [46]. They found that measuring the upper half of the femoral neck with DXA enhanced the prediction of FN hip fracture, while aBMD of the lower half was no different from controls [46]. Alongside recent evidence that osteoporosis treatments and exercise have focal effects on the proximal femoral cortex and trabeculae [27], [47], [48], our CBM results suggest that hip fracture prevention strategies may need to be tailored to improve the focal femoral defects which are characteristic of each fracture type.

There is increased interest in the assessment of CT variables in at-risk patients who are undergoing clinical CT for other diagnostic reasons [49]. CT is also useful for estimating whole bone strength under different simulated loading conditions. Finite element models based on CT data are approved for the clinical prediction of hip fracture and drugs have been shown to strengthen the bone under simulated loading conditions [50], [51]. Several recent studies have used either traditional ROI analyses to investigate the influence of CT cortical bone measures on hip fracture risk [40], [52], [53] or derived novel ROIs using the aforementioned SPM techniques [11], [39], [54] to find 3D bone mineral density patterns associated with hip fracture. All of these have found associations between 3D CT measures and fracture, and our analysis fits well alongside the voxel-based morphometry work of Carballido-Gamio et al. [53]. However, the greater number of fractures of each type (FN and TR) that we had available allowed us to investigate fracture-type specific ROIs. Johannesdottir et al. examined femoral neck cortical thickness in the Icelandic AGES Reykjavik study before Carballido-Gamio et al., but using Mindways CTXA BIT2 software. In that study, the main finding was that supero-anterior femoral neck cortical thickness was strongly related to fracture, especially FN fracture, and remained a significant predictor of hip fracture even after allowing for femoral neck aBMD (also allowing for age, weight and height) [40]. Bousson et al. examined the distribution of CT-derived 3D BMD measures for classifying fracture type. Their results suggested that the volume of cortical bone in the trochanter was the main difference between fracture types (TR and FN) - similar to our findings. In addition, they found that the BMD of the femoral head was a strong predictor of fracture status [39]. With a similar study design to ours, the sensitivity analyses of Bousson are broadly in agreement with our own. Our results, however, pinpoint and quantify precisely where one should look in the 3D structure of the hip as a whole.

The FEMCO protocol also permitted comparison between patients with hip fracture and fallers without hip fracture. Fallers were often frail, and had sustained trauma or even peripheral fractures of one type or another. It was perhaps not surprising (with hindsight) that there were no systemic differences between the hip fracture patients and fallers. Elderly fallers did have focal bone defects in their hips when compared to healthy controls (Fig. 3g) and their future hip fracture risk is presumed to be substantial.

Our study has limitations. Combining analyses of healthy volunteers from several cohorts is a convenience analysis, and neither the sampling of the cases (sequentially during clinical practice) nor controls (sequentially during clinical practice in the UK or via care centres in the Czech Republic) is optimal. Study 1.2 involves subjects from two countries, where either the bone structure of the subjects themselves, the CT scanning technology or calibration differences could introduce systematic errors. To counter this, we modelled study site in all the general linear models. However, when examined on its own, it was clear that there was a systematic difference between values of cortical and trabecular bone between the UK and the Czech Republic. Furthermore, the incidence of hip fracture as well as bone density values differ between Czech Republic and the UK [55]. We suspect that the difference in calibration phantoms (the 5 compartment Mindways K2HPO4 phantom versus the 2 compartment Siemens Osteophantom) may also underlie the systematic difference in CMSD and aBMD that we observed. An additional limitation lies in the fact that the cases and controls from the Czech Republic that we include in the combined analysis here have already been analysed - justified in order to achieve sufficient types of hip fracture, but not optimal. The height and weight measurements were not immediately pre-fracture, being either performed after fracture repair or taken from past GP records. Matching was not achieved in an optimal fashion and application of our results to the general population is not possible from such a sampling method. One subtlety emerges from understanding the potential effects of correlated regressors in SPM, regarding the need to leave out shape mode 1 (overall bone size) and height in the model. This means that the reader must bear in mind that some of the variance in CMSD or ECTD that we show as explained by group (in Fig. 3) might be partly explained by subject height or femur size, since it is well established (and also observed in this study) that larger bones are more susceptible to fracture [11], [56]. Hence a priority is to test 3D bone mapping methods in a study where sampling has been performed appropriately, involving women who have been followed prospectively for incident hip fracture. There were also limitations to the biopsy study; it being performed on a small group of subjects containing a mixture of female and male subjects. While we can use microCT to measure structural parameters, the more traditional technique of histomorphometry may illuminate the remodelling mechanisms for this focal osteoporosis and, for instance, whether fatigue or disuse underpin these focal changes.

## 5 Conclusions

Women who sustain a hip fracture have focal osteoporosis in the proximal femur. Femoral neck and trochanteric hip fractures involve distinct patterns of focal osteoporosis. These findings support the hypothesis that focal osteoporotic defects play a key role in determining fracture risk and fracture location. This information may assist in anatomical targeting of future anti-fracture therapies applied locally through minimally invasive surgery to vulnerable regions of the proximal femur.

## Competing interests

KESP and GMT are inventors on a related patent application GB0917524.1, “Image data processing systems.” This does not alter the authors' adherence to policies on sharing data and materials. KESP has, on behalf of Cambridge Enterprise (CU Enterprise) lectured in educational fora for Amgen and Lilly, he has been an advisory board member for Amgen Inc. and UCB pharma (all honoraria given by CU Enterprise to registered charities) and he has previously received research grant funding from Amgen Inc. and Lilly. GMT has received research grant support from Amgen Inc. and Lilly. AHG has received research grant support from Amgen Inc. and Lilly. TDT has lectured in educational fora for Amgen.

LS, FJ, KEB, CR, MV, SD, JV, PD, MH, JS and JR declare that they have no competing interests.

## Funding information

Arthritis Research UK (grant no. ARC17822) and Cambridge National Institute for Health Research (NIHR) Biomedical Research Centre.

## Figures and Tables

**Table 6:** Odds ratios for hip fracture (discriminating FN or TR fracture from control) per –1SD of variables.

Parameter	Adjusted odds ratio (95%CI) for Trochanteric fx per –1SD	*p* value	Adjusted odds ratio (95%CI) for Femoral neck fx per -1SD)	*p* value
DXA-like Fn aBMD	2.543 (1.788 to 3.617) 3.363 (2.206 to 5.129) 2.566 (1.737 to 3.792) 2.161 (1.498 to 3.118) 4.099 (2.650 to 6.339) 4.196 (2.703 to 6.513)	< 0.00001	2.567 (1.876 to 3.513) 1.453 (1.091 to 1.935) 1.841 (1.340 to 2.529) 2.53 (1.813 to 3.531) 1.758 (1.317 to 2.347) 2.575 (1.848 to 3.587)	< 0.00001
DXA-like Th aBMD		< 0.00001		0.00918
CMSD patch (Trochanteric Fig. 3d)		< 0.00001		0.00012
CMSD patch (Femoral neck Fig. 3c)		0.00003		< 0.00001
ECTD patch (Trochanteric Fig. 3f)		< 0.00001		0.00009
ECTD patch (Femoral neck Fig. 3e)		< 0.00001		< 0.00001

**Table 1:** Key protocol details for FEMCO Study (LREC07/H0305/61).

Protocol title, REC number, REC committee	Regional thinning of the FEMoral neck COrtex in hip fracture; a case-control study LREC07/H0305/61 ARC17822 v3.6 Cambridge Research Ethics Committee 4
Objective	To evaluate a novel index of bone fragility (regional cortical thickness) using clinical quantitative computed tomography (QCT) scanning of the proximal femur
Study design	Case control study, convenience sampling for cases and controls
Setting	Multicentre, UK (Cambridge, Norwich, Torbay). Initiated in 2007
Participants	Eligibility- inclusion criteria. Cases Patients with first hip fracture (femoral neck or trochanteric) awaiting surgical fixation, not due for surgery within 4 h of consent, able to understand, ask questions and give witnessed consent (verbal or written), medically stabilised. Controls Patients with a recent admission to orthogeriatric unit following fall from standing height or less, not sustaining hip fracture. Exclusion criteria- Dementia/cognitive impairment (AMTS < 7/10 or MMSE < 19/30), unconsciousness, terminal illness, metastatic cancer, previous hip replacement (synthetic material at either hip), previous hip fracture, osteomyelitis, bone tumour, currently taking oral corticosteroids, prior hemiplegia, prior treatment with teriparatide or strontium ranelate
Matching criteria	Convenience sample of cases. Convenience sample of fallers, not matched beyond sex, minimum age and injurious fall.
Scan protocol	Patient Positioning for Hip QCT Supine on Siemens Somatom Sensation 16, 64 or GE Lightspeed 64 scanner. Mindways 5-compartment solid phantom positioned under the hips (calibrated to aqueous K2HPO4 density), or phantom-free (using ClinicQCT asynchronous calibration) if phantom previously calibrated on that machine. Acquisition parameters: scout view from iliac crest to lesser trochanters. 120 kV, Tube current target 160mAS with Siemens CARE dosing, GE target dosing up to 320mAS. Reconstruction: To capture both hips and the phantom in each reconstruction 1 mm slice thickness (0.5 mm increment) on Siemens or 1.25 mm (0.625 increment) on GE 64. DFOV 400 mm (512*512 pixel matrix) = pixel size 0.5859 mm. Siemens B20f convolution kernel, GE ‘bone’ kernel. CT DICOM format images. Fracture classification: a consultant radiologist (TDT) reconstructed CT images with a multiplanar reformat (MPR) and classified the fracture as subcapital, transcervical or trochanteric using the Muller AO classification, based on the anterior extent of the fracture line. Image processing: QCT PRO CTXA software (v5.1.3) - reconstruct 3D image, analyse each hip for vBMD, aBMD. Segmentation of contralateral hip in fracture patients (Stradwin v 4.0) or both hips in control patients followed by Bone Mapping.
Participants used for present analysis (n)	Study 1.1 Hip fracture cases 50 females (37 femoral neck, 13 trochanteric) Study 1.1 Hip fracture cases 20 males (14 femoral neck, 6 trochanteric) Study 1.2 Hip fracture cases 60 females (46 femoral neck, 14 trochanteric) Study 1.3 Frail fallers 50 females
Demographics	Fall description, site of impact, other injuries, admitted from home/institution, MUST category (malnutrition index), weight, last recorded height (either GP record or measured), FRAX questions, pre admission Barthel Index and Functional Ambulatory Category, bone active medications, EPOS hip questionnaire.
Bone density analysis	aBMD of the femoral neck and total hip region region using traditional ROIs specified in CTXA software (QCTpro v 5.1.3).

**Table 2:** Key protocol details for Anglo-Cardiff Collaborative Trial (LREC 04/Q0108/257).

Protocol title, REC number, REC committee	Isolated systolic hypertension, arterial stiffening and calcification LREC04/Q0108/257 Cambridge Research Ethics Committee 4.
Objective	To compare arterial stiffening with bone density in healthy subjects using clinical quantitative computed tomography (QCT) scanning of the proximal femur
Study design	Case Control study, at random from local general practice lists by letter of invitation (the overall response rate was 85%).
Setting	Multicentre, UK (Cambridge and Cardiff) Initiated in 2005
Participants	Eligibility- Inclusion criteria. Controls Healthy males not taking any medication able to understand, ask questions and give witnessed consent (verbal or written), medically stabilised. Exclusion criteria- Diabetes mellitus, hypercholesterolemia (self-reported or total cholesterol ≥ 6.5 mmol/L), renal disease (defined as a clinical history, creatinine ≥ 150 umol/L, or an active urinary sediment), a history of cardio- vascular disease (defined as a clinical history or evidence on examination), known inflammatory conditions, malignancy, or a recent history of infection, dementia/cognitive impairment (AMTS < 7/10 or MMSE < 19/30), unconsciousness, terminal illness, metastatic cancer, previous hip replacement (synthetic material at either hip), previous hip fracture, osteomyelitis, bone tumour.
Matching criteria	Not matched beyond sex and minimum age.
Scan protocol	Patient Positioning for Hip QCT Supine on Siemens Somatom Sensation 16 scanner. Mindways 5-compartment solid phantom positioned under the hips (calibrated to aqueous K2HPO4 density) Acquisition parameters: Scout view from iliac crest to lesser trochanters. 120 kV, Tube current target 160mAS with Siemens CARE dosing Reconstruction: To capture both hips and the phantom in each reconstruction 1 mm slice thickness (0.5 mm increment) on Siemens DFOV 400 mm (512 ∗ 512 pixel matrix) = pixel size 0.5859 mm. Siemens B20f convolution kernel. CT DICOM format images. Image Processing: QCT PRO CTXA software (v5.1.3) - Reconstruct 3D image, analyse each hip for vBMD, aBMD. Segmentation of contralateral hip in fracture patients (Stradwin v 4.0) or both hips in control patients followed by Bone Mapping.
Participants used for present analysis (n)	Study 1.2 Healthy controls 20
Demographics	Weight, last recorded height (either GP record or measured).
Bone density analysis	aBMD of the femoral neck and total hip region region using traditional ROIs specified in CTXA software (QCTpro v 5.1.3).

**Table 3:** Key protocol details for Cambridge MRC-Ageing study (LREC 06/Q0108/180).

Protocol title, REC number, REC committee	Cortical thinning measured in vivo: determinant Of hip fracture risk MRC LREC 06/Q0108/180 G0501550 version 1.0. Cambridge Research Ethics Committee
Objective	To determine how accurately cortical thickness in the femoral neck can be measured in vivo with the purpose of determining changes with age and whether this approach increases the ability to detect those at increased risk of hip fracture
Study design	Observational study of ageing, convenience sampling for participants
Setting	Single centre, Cambridge UK. Recruitment started in 2003
Participants	Eligibility - inclusion criteria. Female, age over 20, healthy volunteers attending Addenbrooke's NHS Trust for a routine clinical CT scan which includes the abdomen and pelvis Exclusion criteria- Dementia/cognitive impairment, unconsciousness, terminal illness, metastatic cancer, previous hip replacement (synthetic material at either hip), previous hip fracture, osteomyelitis, bone tumour, known history of metabolic bone disease, those taking oral corticosteroids, women already enrolled in a study involving X-rays.
Participants used for present analysis (n)	Study 1.2 and 1.3 Healthy controls 32
Scan protocol	See FEMCO study. Not using GE Lightspeed scanner. Further details in journal article [24]
Bone density analysis	aBMD of the femoral neck and total hip region region using traditional ROIs specified in CTXA software (QCTpro v 5.1.3).

**Table 4:** Key protocol details for Cambridge MRC-Hip Fx study (LREC 99/076).

Protocol title, REC number, REC committee	Measurement of femoral neck bone loss in cases of hip fracture compared to hospital controls MRC LREC99/076 version 1.0. Cambridge Research Ethics Committee
Objective	To estimate cortical bone stability in the contralateral hip of fracture cases compared with measurements of age matched controls
Study design	Case Control study, convenience sampling for cases and controls
Setting	Single centre, Cambridge, UK. Recruitment started in 2001
Participants	Eligibility - inclusion criteria. Cases Female with first hip fracture (femoral neck or trochanteric) post surgical fixation, able to understand, ask questions and give witnessed consent, medically stabilised. Controls healthy volunteers attending Addenbrooke's NHS Trust for a routine clinical CT scan which includes the abdomen and pelvis, who were subsequently found to have no carcinoma. Exclusion criteria- Dementia/cognitive impairment, unconsciousness, terminal illness, metastatic cancer, previous hip replacement (synthetic material at either hip), previous hip fracture, osteomyelitis, bone tumour, known history of metabolic bone disease, those taking oral corticosteroids, women already enrolled in a study involving x-rays.
Participants used for present analysis (n)	Study 1.2 and 1.3 healthy controls 18
Scan protocol	See FEMCO study. Not using GE Lightspeed scanner
Bone density analysis	aBMD of the femoral neck and total hip region region using traditional ROIs specified in CTXA software (QCTpro v 5.1.3)

**Table 5:** Key protocol details for Prague Study of Hip Joint in Trauma study.

Protocol title, REC number, REC committee	Study of Hip Joint in Trauma IRB0002384101 (Ethical Committee of the Institute of Rheumatology and Ethical Committee of Bulovka Hospital). FEMCO amendment (approved by Cambridgeshire LREC4)
Objective	To measure bone density by QCT in the femoral head of fracture cases compared with age matched controls.
Study design	Case Control study, convenience sampling for cases and controls
Setting	Single centre, Prague, Czech Republic. Recruitment started in 2006
Participants	Eligibility- inclusion criteria. Cases Female with first hip fracture (femoral neck or trochanteric) attending Bulovka University Hospital Prague, awaiting surgical fixation, able to give informed consent, low energy injury. Controls healthy volunteers by invitation at rheumatology clinics and two residential care centres in the same districts of Prague, attending Homolka Hospital Prague. Exclusion criteria- terminal illness, metastatic cancer, previous hip replacement (synthetic material at either hip), subtrochanteric fracture, known history of unilateral bone disease.
Participants used for present analysis (n)	Study 1.2 Hip fracture cases 78 (40 femoral neck 38 trochanteric) Study 1.2 Control fallers 75
Scan protocol	Patient Positioning for Hip QCT Supine on Siemens Somatom Sensation 16 or 40 scanner. Siemens two-compartment Osteo phantom. Reconstruction: To capture both hips and the phantom in each reconstruction. Siemens B10/B20s convolution kernel, ≤ 1 mm reconstructed slice thickness CT DICOM format images. Fracture classification: A consultant radiologist (TDT) reconstructed CT images with a multiplanar reformat (MPR) and classified the fracture as subcapital, transcervical or trochanteric using the Muller AO classification, based on the anterior extent of the fracture line. Image Processing: QCT PRO CTXA software (v5.1.3)- Reconstruct 3D image, analyse each hip for vBMD, aBMD. Segmentation of contralateral hip in fracture patients (Stradwin v 4.0) or both hips in control patients followed by Bone Mapping. Further details in journal article [6]
Bone density analysis	aBMD of the femoral neck and total hip region region using traditional ROIs specified in CTXA software (QCTpro v 5.1.3).

## References

[1] Parker M., Johansen A. (2006). Hip fracture. BMJ.

[2] Clinical Guideline (CG124) (2011). The management of hip fracture in adults. Excellence NIfHaC.

[3] Pulkkinen P., Gluer C.C., Jamsa T. (2011). Investigation of differences between hip fracture types: a worthy strategy for improved risk assessment and fracture prevention. Bone.

[4] Moja L., Piatti A., Pecoraro V. (2012). Timing matters in hip fracture surgery: patients operated within 48 hours have better outcomes. A meta-analysis and meta-regression of over 190,000 patients. PLoS One.

[5] Juszczyk M.M., Cristofolini L., Salva M., Zani L., Schileo E., Viceconti M. (2013). Accurate in vitro identification of fracture onset in bones: failure mechanism of the proximal human femur. J. Biomech..

[6] Schileo E., Balistreri L., Grassi L., Cristofolini L., Taddei F. (2014). To what extent can linear finite element models of human femora predict failure under stance and fall loading configurations?. J. Biomech..

[7] de Bakker P.M., Manske S.L., Ebacher V., Oxland T.R., Cripton P.A., Guy P. (2009). During sideways falls proximal femur fractures initiate in the superolateral cortex: evidence from high-speed video of simulated fractures. J. Biomech..

[8] Mayhew P.M., Thomas C.D., Clement J.G. (2005). Relation between age, femoral neck cortical stability, and hip fracture risk. Lancet.

[9] Issever A.S., Burghardt A., Patel V. (2003). A micro-computed tomography study of the trabecular bone structure in the femoral head. J. Musculoskelet. Neuronal Interact..

[10] Jenkins P.J., Ramaesh R., Pankaj P. (2013). A micro-architectural evaluation of osteoporotic human femoral heads to guide implant placement in proximal femoral fractures. Acta Orthop..

[11] Carballido-Gamio J., Harnish R., Saeed I. (2013). Proximal femoral density distribution and structure in relation to age and hip fracture risk in women. J. Bone Miner. Res..

[12] Nicolella D.P., Bredbenner T.L. (2012). Development of a parametric finite element model of the proximal femur using statistical shape and density modelling. Comput. Methods Biomech. Biomed. Engin..

[13] Treece G.M., Poole K.E., Gee A.H. (2012). Imaging the femoral cortex: thickness, density and mass from clinical CT. Med. Image Anal..

[14] Treece G.M., Gee A.H., Mayhew P.M., Poole K.E. (2010). High resolution cortical bone thickness measurement from clinical CT data. Med. Image Anal..

[15] Carballido-Gamio J., Nicolella D.P. (2013). Computational anatomy in the study of bone structure. Curr. Osteoporos. Rep..

[16] Carballido-Gamio J., Harnish R., Saeed I. (2013). Structural patterns of the proximal femur in relation to age and hip fracture risk in women. Bone.

[17] Poole K.E., Treece G.M., Mayhew P.M. (2012). Cortical thickness mapping to identify focal osteoporosis in patients with hip fracture. PLoS One.

[18] Nawathe S., Akhlaghpour H., Bouxsein M.L., Keaveny T.M. (2014). Microstructural failure mechanisms in the human proximal femur for sideways fall loading. J. Bone Miner. Res..

[19] Bessho M., Ohnishi I., Matsumoto T. (2009). Prediction of proximal femur strength using a CT-based nonlinear finite element method: differences in predicted fracture load and site with changing load and boundary conditions. Bone.

[20] Treece G.M., Gee A.H., Tonkin C. (2015). Predicting hip fracture type with cortical bone mapping (CBM) in the osteoporotic fractures in men (MrOS) study. J. Bone Miner. Res..

[21] Seeherman H.J., Li X.J., Smith E., Parkington J., Li R., Wozney J.M. (2013). Intraosseous injection of rhBMP-2/calcium phosphate matrix improves bone structure and strength in the proximal aspect of the femur in chronic ovariectomized nonhuman primates. J. Bone Joint Surg. Am..

[22] Cann C.E., Adams J.E., Brown J.K., Brett A.D. (2014). CTXA hip–an extension of classical DXA measurements using quantitative CT. PLoS One.

[23] Treece G.M., Gee A.H. (2015). Independent measurement of femoral cortical thickness and cortical bone density using clinical CT. Med. Image Anal..

[24] Poole K.E., Mayhew P.M., Rose C.M. (2010). Changing structure of the femoral neck across the adult female lifespan. J. Bone Miner. Res..

[25] McEniery C.M., McDonnell B.J., So A. (2009). Aortic calcification is associated with aortic stiffness and isolated systolic hypertension in healthy individuals. Hypertension.

[26] Muller M.E. (1980). Classification and international AO-documentation of femur fractures. Unfallheilkunde.

[27] Poole K.E., Treece G.M., Gee A.H. (2015). Denosumab rapidly increases cortical bone in key locations of the femur: A 3D bone mapping study in women with osteoporosis. J. Bone Miner. Res..

[28] Whitmarsh T., Fritscher K.D., Humbert L. (2011). A statistical model of shape and bone mineral density distribution of the proximal femur for fracture risk assessment. Medical image computing and computer-assisted intervention.

[29] Whitmarsh T., Humbert L., De Craene M., Del Rio Barquero L.M., Frangi A.F. (2011). Reconstructing the 3D shape and bone mineral density distribution of the proximal femur from dual-energy X-ray absorptiometry. IEEE Trans. Med. Imaging.

[30] Friston K.J., Holmes A.P., Worsley K.J., Poline J.-P., Frith C.D., Frackowiak R.S.J. (1994). Statistical parametric maps in functional imaging: A general linear approach. Hum. Brain Mapp..

[31] Worsley K., Taylor J., Carbonell F. (2009). SurfStat: a Matlab toolbox for the statistical analysis of univariate and multivariate surface and volumetric data using linear mixed effects models and random field theory. NeuroImage Organization for Human Brain Mapping 2009 Annual Meeting.

[32] Gee A.H., Treece G.M. (2014). Systematic misregistration and the statistical analysis of surface data. Med. Image Anal..

[33] Andrade A., Paradis A.L., Rouquette S., Poline J.B. (1999). Ambiguous results in functional neuroimaging data analysis due to covariate correlation. NeuroImage.

[34] Poline J., Kherif F., Pallier C., Penny W., Friston K.J., Ashburner J.T., Kiebel S., Nichols T., Penny W.D. (2007). Contrasts and classical inference. Statistical Parametric Mapping: The Analysis of Functional Brain Images.

[35] Doube M., Klosowski M.M., Arganda-Carreras I. (2010). BoneJ: Free and extensible bone image analysis in ImageJ. Bone.

[36] Schwartzbaum J., Ahlbom A., Feychting M. (2003). Berkson's bias reviewed. Eur. J. Epidemiol..

[37] Thomas C.D., Mayhew P.M., Power J. (2009). Femoral neck trabecular bone: loss with aging and role in preventing fracture. J. Bone Miner. Res..

[38] Huber M.B., Carballido-Gamio J., Bauer J.S. (2008). Proximal femur specimens: automated 3D trabecular bone mineral density analysis at multidetector CT–correlation with biomechanical strength measurement. Radiology.

[39] Bousson V.D., Adams J., Engelke K. (2011). In vivo discrimination of hip fracture with quantitative computed tomography: results from the prospective European Femur Fracture Study (EFFECT). J. Bone Miner. Res..

[40] Johannesdottir F., Poole K.E., Reeve J. (2011). Distribution of cortical bone in the femoral neck and hip fracture: a prospective case-control analysis of 143 incident hip fractures; the AGES-REYKJAVIK Study. Bone.

[41] Issever A.S., Walsh A., Lu Y., Burghardt A., Lotz J.C., Majumdar S. (2003). Micro-computed tomography evaluation of trabecular bone structure on loaded mice tail vertebrae. Spine.

[42] Milovanovic P., Djonic D., Marshall R.P. (2012). Micro-structural basis for particular vulnerability of the superolateral neck trabecular bone in the postmenopausal women with hip fractures. Bone.

[43] Chiba K., Burghardt A.J., Osaki M., Majumdar S. (2013). Heterogeneity of bone microstructure in the femoral head in patients with osteoporosis: an ex vivo HR-pQCT study. Bone.

[44] Homminga J., McCreadie B.R., Ciarelli T.E., Weinans H., Goldstein S.A., Huiskes R. (2002). Cancellous bone mechanical properties from normals and patients with hip fractures differ on the structure level, not on the bone hard tissue level. Bone.

[45] Albright F., Reifenstein J.E.C. (1948). The Parathyroid Glands and Metabolic Bone Disease – Selected Studies.

[46] Duboeuf F., Hans D., Schott A.M. (1997). Different morphometric and densitometric parameters predict cervical and trochanteric hip fracture: the EPIDOS Study. J. Bone Miner. Res..

[47] Poole K.E., Treece G.M., Ridgway G.R., Mayhew P.M., Borggrefe J., Gee A.H. (2011). Targeted regeneration of bone in the osteoporotic human femur. PLoS One.

[48] Allison S.J., Poole K.E., Treece G.M. (2015). The influence of high-impact exercise on cortical and trabecular bone mineral content and 3D distribution across the proximal femur in older men: a randomized controlled unilateral intervention. J. Bone Miner. Res..

[49] Pickhardt P.J., Pooler B.D., Lauder T., del Rio A.M., Bruce R.J., Binkley N. (2013). Opportunistic screening for osteoporosis using abdominal computed tomography scans obtained for other indications. Ann. Intern. Med..

[50] Kopperdahl D.L., Aspelund T., Hoffmann P.F. (2014). Assessment of incident spine and hip fractures in women and men using finite element analysis of CT scans. J. Bone Miner. Res..

[51] Keaveny T.M., McClung M.R., Genant H.K. (2014). Femoral and vertebral strength improvements in postmenopausal women with osteoporosis treated with denosumab. J. Bone Miner. Res..

[52] Black D.M., Bouxsein M.L., Marshall L.M. (2008). Proximal femoral structure and the prediction of hip fracture in men: a large prospective study using QCT. J. Bone Miner. Res..

[53] Lang T.F., Sigurdsson S., Karlsdottir G. (2012). Age-related loss of proximal femoral strength in elderly men and women: the age gene/environment susceptibility study — Reykjavik. Bone.

[54] Li W., Kornak J., Harris T. (2009). Identify fracture-critical regions inside the proximal femur using statistical parametric mapping. Bone.

[55] Cauley J.A., Chalhoub D., Kassem A.M., Fuleihan G.E. (2014). Geographic and ethnic disparities in osteoporotic fractures. Nat. Rev. Endocrinol..

[56] Whitmarsh T., Fritscher K.D., Humbert L. (2012). Hip fracture discrimination from dual-energy X-ray absorptiometry by statistical model registration. Bone.

